# Retinal Vessel Extraction *via* Assisted Multi-Channel Feature Map and U-Net

**DOI:** 10.3389/fpubh.2022.858327

**Published:** 2022-03-17

**Authors:** Surbhi Bhatia, Shadab Alam, Mohammed Shuaib, Mohammed Hameed Alhameed, Fathe Jeribi, Razan Ibrahim Alsuwailem

**Affiliations:** ^1^Department of Information Systems, College of Computer Sciences and Information Technology, King Faisal University, Hofuf, Saudi Arabia; ^2^College of Computer Science and Information Technology, Jazan University, Jazan, Saudi Arabia

**Keywords:** multichannel, retinal vessels, retinopathy, U-Net, Cauchy distribution

## Abstract

Early detection of vessels from fundus images can effectively prevent the permanent retinal damages caused by retinopathies such as glaucoma, hyperextension, and diabetes. Concerning the red color of both retinal vessels and background and the vessel's morphological variations, the current vessel detection methodologies fail to segment thin vessels and discriminate them in the regions where permanent retinopathies mainly occur. This research aims to suggest a novel approach to take the benefit of both traditional template-matching methods with recent deep learning (DL) solutions. These two methods are combined in which the response of a Cauchy matched filter is used to replace the noisy red channel of the fundus images. Consequently, a U-shaped fully connected convolutional neural network (U-net) is employed to train end-to-end segmentation of pixels into vessel and background classes. Each preprocessed image is divided into several patches to provide enough training images and speed up the training per each instance. The DRIVE public database has been analyzed to test the proposed method, and metrics such as Accuracy, Precision, Sensitivity and Specificity have been measured for evaluation. The evaluation indicates that the average extraction accuracy of the proposed model is 0.9640 on the employed dataset.

## Introduction

Retinal vessel detection and identifying blood vessel properties, including diameter, shape, bifurcation, and tortuosity, mainly aim to diagnose various eye abnormalities and retinopathies (1). At the same time, the evaluation of bifurcation points and tortuosity assist in diagnosing cardiovascular ailments. Also, analyzing width can result in the prevention of retinopathies induced by hypertension (2). Besides, glaucoma prevention is only possible if fundus retinal images are analyzed regularly to diagnose any abnormal modification of blood vessels around the optic disk (3). Diabetic retinopathy is another disease imposed by blood vessel construction, distribution changes, and fresh vessel evolution. Late diagnosis may also result in adult blindness (4). These critical medical applications highlight the importance of vessel extrication from retinal fundus pictures for ophthalmologists in diagnosing diseases and acting properly for their patients.

Unfortunately, with such a significant role the retinal vessel detection, blood vessels are not visible easily in retinal images taken by the fundus. The red background of the retina alongside the red color of retinal vessels is combined with fading small vessels spread around the retina. Therefore, manual detection of blood vessels is considered a subjective task requiring specific expertise while time-consuming (5). The vessel's width varies subject to the breadth of the vessel and the image resolution. The retinal boundary, optic disc, and diseases such as cotton wool patches, bright and black lesions, and exudate can all be seen in ocular fundus images (3).

Image processing methods have improved the recognition of blood vessels by changing the intensity of images and normalizing the color distribution. These techniques can enhance the reading of retinal images; however, fully automatic extraction requires understanding the properties of the vessel and detecting them in the noisy background of retinal images at which inadequate contrast regions are distrusted non-uniformly. It has been more than three decades where Computer-Aided-Detection (CAD) methods have been employed to diagnose retinopathies. Unfortunately, most traditional methods are not well-suited for recent, accurate sensors. For instance, since the resolution of fundus scanners is increased, standard image-processing methods show their drawback in detecting small faded vessels. Briefly said, the traditional systems underperform the detection due to variations in image properties due to different capture devices that influence the intensity and resolution of retinal images; therefore, machine learning-based approaches are more suitable (6).

For vascular segmentation of retinal pictures, various machine learning (ML) approaches and procedures are available. These methods are divided into supervised and unsupervised approaches. Support vector machine (SVM), neural network, and classification based methods are examples of supervised methods. Matching filter, vessel tracking, and mathematical morphology methods are all examples of unsupervised approaches (7).

The remaining current paper is divided into three sections. In Section Literature Review, a brief review will be conducted on the approaches targeting the objective of the work *via* various techniques. The section is wrapped up by highlighting the importance of fusion approaches taking novel DL methods with successful traditional approaches at once. In the third section, the main idea of the research is proposed, while the user data set and the structure of the employed DL is detailed. Later in Section Results and Discussions, results will be reported, and evaluations will be carried out to identify the strengths and limitations of the suggested approaches compared with the other similar methods. The conclusion is then followed in Section Conclusion and Future Work to wrap up the discussed methodology.

## Literature Review

**Figure 1:**
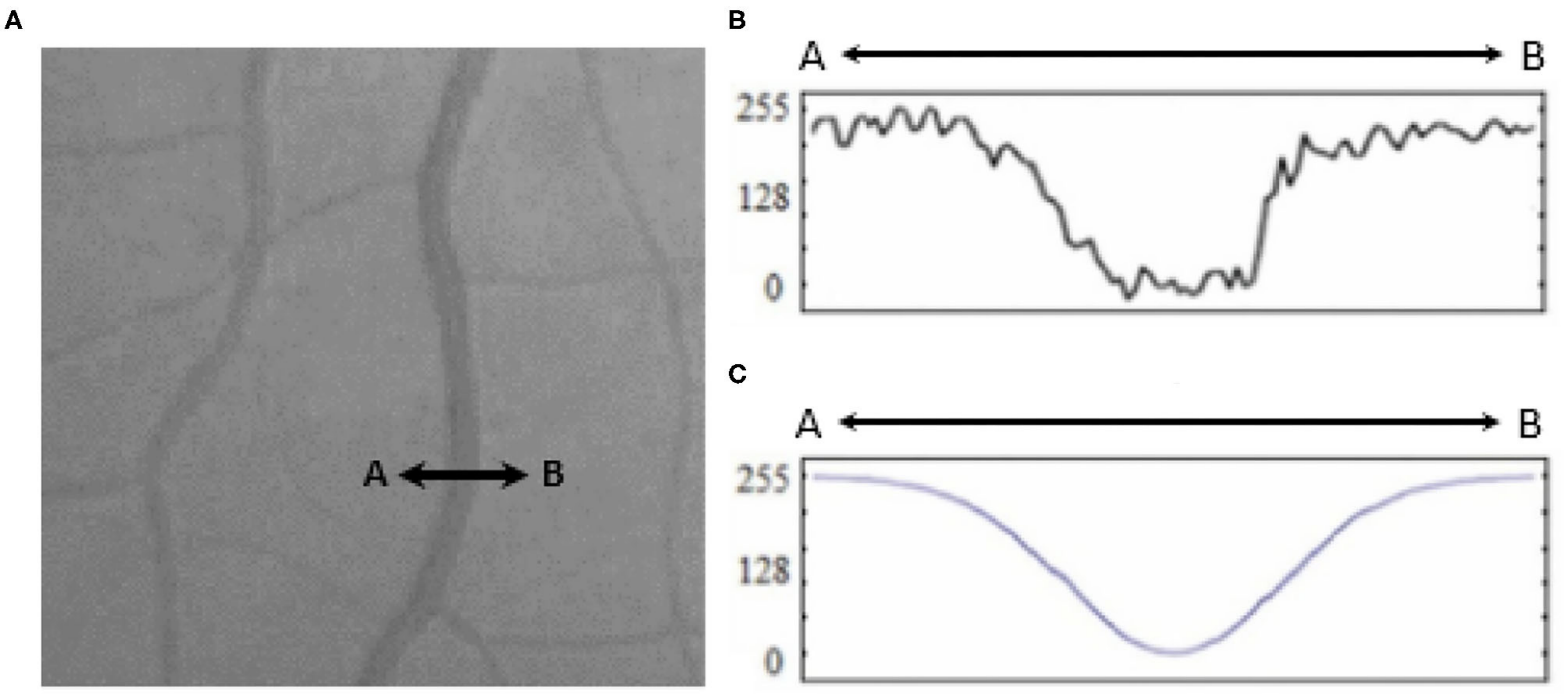
**(A)** Retinal vessel cross-section (A to B), **(B)** intensities of the cross-section, and **(C)** fitted Gaussian curve proposed by Chaudhury et al. (9).

A broad look at the Literature review covering retinal blood vessel detection methodologies reveals that the current methodologies can be categorized into three classes (8) template matching based, tracking-based, and classifying based. In template matching approaches, a kernel is selected, which can be fitted well to a vessel. This kernel is later convoluted with each image. At the final stage, pixels with the highest responses are collected *via* a proper thresholding method to provide a binary map of vessel/background. A Gaussian-shaped curve proposed by Chaudhuri et al. (9) is used to highlight the vessels based on the intensity distribution of the vessel's cross-section (as illustrated in Figure 1).

Later improvement has been proposed by Al-Rawi et al. (10) to tune the parameters to increase the Sensitivity and Accuracy and decrease false positive detection. Gaussian, Cauchy, and Gabor distributions have been employed with the same purpose (3).

In contrast to the previous two approaches in which a template of the vessels plays an essential role in the detection, tracking approaches use a model to trace vessels *via* seed growing. Each of the vessels can be traced separately from a starting point (which can be an ending point of the parent vessel) to the ending point at which the vessel is branched. Considering the contrast between vessels and other retina background intensity, Wu et al. (11) determined the mid-line of a vessel from a starting point inside it. Having calculated the vessel's width, tracing the orientation is taken into place automatically by assuming the vessel boundaries are parallel with the middle line. Several discussions have been conducted to detect the initial seed point *via* manual annotation or automatic template matching methods (7). For instance, the methodology proposed by Dizdaro et al. used level-set thresholding to identify an initial seed (12). The work carried out by Zhang et al., which applied active contours started from a seed found *via* Hessian vessel response (13), can be categorized in the second explained group using a tracking approach to detect vessels. Ali et al. used B-COSFIRE filter blending with adaptive thresholding for binarization of retinal blood vessels (14).

In classifying approaches, mostly ML techniques are taken into consideration. ML methods focus on two sets of features: common between pixels belonging to vessels and those that discriminate between background and vessel pixels. Provision of such pixels can increase the accuracy of a model being trained by artificial intelligence (AI) (15, 16).

On the one hand, practical ML approaches with supervised methods target pixel-based feature maps as training to identify vessel patterns. Various methodologies have been proposed to detect vessels using k-Nearest Neighbors (17), Decision Trees (18), SVM (19), and Neural Networks (20, 21). On the other hand, unsupervised methods employ rule-based algorithms, including filters, gradients, and thresholds for the same classification purpose. For instance, the work presented in (22) employs a neural network trained to model a matched filter.

Traditional ML algorithms and recent deep-learning methods can be listed as the third set of approaches. Recent studies on DL comprise two perspectives, in which the first one addresses retinal vessel detection *via* pixel-level classification. The later perspective uses DL to achieve semantic segmentation between the two classes of pixels. An instance can be the pipeline of methodologies proposed by Wang et al. (23). They extract a set of required features using DL and latterly distinguish each pixel *via* Random Forests.

Ensemble learning has also been used in several investigations. Lahiri et al. (24) suggested using an ensemble of stacked denoising autoencoders. Subsequently, the final conclusion is made *via* voting of a SoftMax layer over autoencoder outputs. In another work, Maji et al. (25) suggested an ensemble of 12 deep Convolutional neural networks (CNN). The final decision is made *via* performing averaging of the output result of the decision over each pixel. Few studies have targeted DNN and formulated the objective into a pixel-to-pixel classification (26, 27); however, these approaches are underperforming the performance by picking each pixel and deciding the proper class in terms of training or testing. A practical solution has been proposed by Dasgupta et al. (28) in which both training and testing phases are carried out over small retinal patches with an end-to-end perspective. These patches are individual from each other, and hence they can be processed simultaneously.

Wu et al. (29) suggested employing a CNN to extract binary masks followed by a generalized particle filtering technique aiming at retinal vessel tree extraction *via* a probabilistic tracking framework. Concerning long-range collaborations between pixels, Fu et al. (30) carried out the vessel detection by Conditional Random Field (CRF) developed based on a multi-level CNN model.

**Figure 2:**
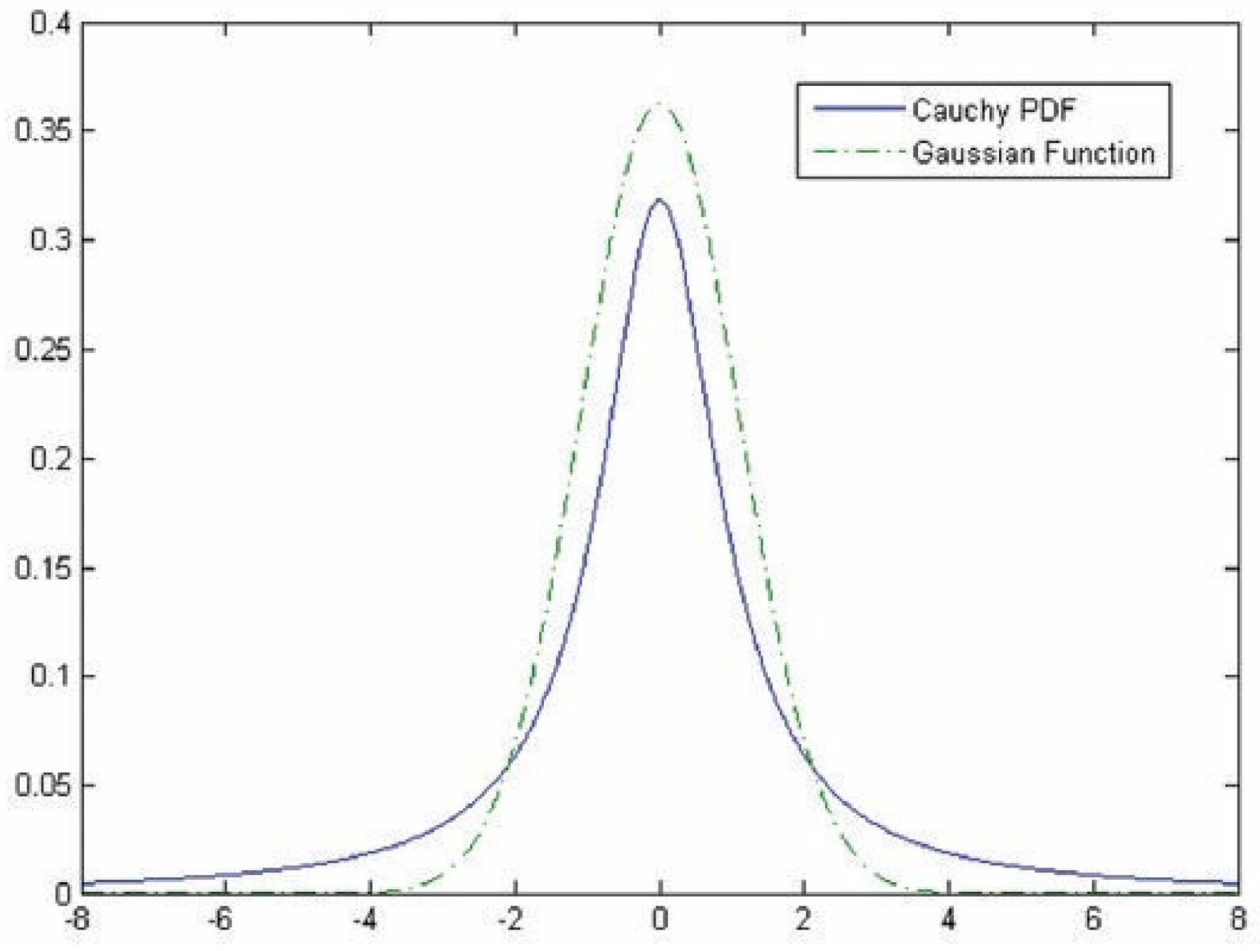
Comparison of the Gaussian and the Cauchy curves for generating a template for retinal vessels [The image is adapted from (34)].

Hybrid methodologies can be suggested *via* a combination of the approaches mentioned above. For instance, the template matching approach can be utilized in the third approach to provide more features for classifying vessels. Studies combine these approaches in sequential order, as template matching has been considered in the preprocessing stage to highlight features that will be used later by the ML approach to discriminate pixels belonging to either retinal tissue or vessel (31, 32). For instance, the work proposed by Gao et al. (33) combines Gaussian matched filter (as a preprocessing stage) with a U-Net CNN to improve vessel detection. In other studies, Zolfagharnasab et al. (34) showed that the Cauchy probability distribution function (PDF) could be a model cross-section of vessels more accurate than the Gaussian. The comparison of Gaussian and the Cauchy curves for generating a template for retinal vessels has been shown in Figure 2.

In this way, the conducted study aims to suggest two main contributions in the pipeline of the work proposed by Gao et al. First, supported by the investigation conducted in (33), we will employ Cauchy PDF instead of Gaussian. Second, a novel feature map will be prepared instead of raw pixel intensities by proposing a new three-channel image. Roy and Sharma presented a Residual Y-net design for retinal vessel detection inspired by U-net that help in Diabetic detection (35). Siddique et al. gave a review of U-Net and its variant techniques for image segmentation and specifically highlighted their applications in retinal fundus image segmentation (36). Yuliang et al. propose a retinal blood vessel segmentation model that combines a multiscale matched filter with a U-Net that has been tested on various available public datasets (37). Shabbir et al. describe different types of ML models for glaucoma detection from fundus image like multiscale, texture feature-based, Segmentation-based, CNN, Ensemble Learning approaches in detail (38). Wang et al. proposed a structure using Dense U-net and the patch-based learning approach for clinical applications (39). Table 1 given below provides a tabular representation of the related work discussed in the literature review section.

**Table 1:** Related works.

**References**	**Used techniques**	**Main idea of paper**
(24)	Unsupervised hierarchical feature learning and ensemble	Suggested using an ensemble of stacked denoising autoencoders. Subsequently, the final decision was made *via* voting of a SoftMax layer over autoencoder outputs.
(29)	Deep CNN and nearest neighbor search	Suggested the employment of CNN to extract binary masks followed by a generalized particle filtering technique for vascular structure identification from medical images aiming at retinal vessel tree extraction *via* a probabilistic tracking structure.
(30)	Conditional Random Field (CRF), multi-level CNN	Carried out the vessel detection by Conditional Random Field (CRF) developed based on a multi-level CNN model.
(34)	Cauchy PDF and Gaussian function	Showed that the Cauchy probability distribution function (PDF) could be a model cross-section of vessels more accurate than the Gaussian.
(33)	Gaussian matched filter with U-Net	To use Gaussian matched filter in the preprocessing stage with U-Net CNN, aiming to improve the detection of vessels.
(35)	Y-net	Presented a Residual Y-net design for retinal vessel detection inspired by U-net that help in Diabetic detection.
(36)	U-Net	A review of U-Net and its variant techniques for image segmentation and specifically highlight their applications in retinal fundus image segmentation.
(37)	Multiscale matched filter, U-Net	Proposes a model that combines a multiscale matched filter with a U-Net that has been tested on various available public datasets.
(38)	Multiscale, texture feature-based, Segmentation-based, CNN, Ensemble Learning approaches	Describe different types of ML models for glaucoma detection from fundus images like multiscale, texture feature-based, Segmentation-based, CNN, Ensemble Learning approaches in detail.
(39)	Dense U-net	Proposed a structure using Dense U-net and the patch-based learning approach for clinical applications.

## Methodology

The end-to-end implementation of retinal vessel detection requires introducing a correct set of features per pixel in which the model can find meaningful discrimination between vessels and background. Concerning the intensity of each pixel in the fundus image, *I*(*x, y*) can be defined where x and y are the pixel intensity, and *I* is the content of the pixel intensity reported in the RGB vector. Several studies have suggested a preprocessing step to enhance the brightness of fundus images (29, 40).

### Pre-processing

Illustrated in Figure 1, the original fundus images present low-intensity differences among the retina tissue and vessels; therefore, it is proposed to use contrast limited adaptive histogram equalization operation (known as CLAHE) to improve the overall image distinction (1). The CLAHE operation was performed for each color channel to normalize the pixel intensity values image *via* (1).


(1) $$\begin{array}{r} I\left(x,y\right)=\frac{I\left(x,y\right)-u}{\sigma } \end{array}$$

Where μ is mean, and σ is the standard deviation of each channel.

Concerning the red nature of blood vessels, since the retina texture is supplied *via* micro-vessels, the mean intensity of the red channel is mostly higher than other RGB channels. To this end, it has been reported to remove the red channel from the processing of retinal vessels. In contrast, the green channel and the blue one can be used instead to provide more information about the spread of blood vessels. To this end, an initial step comprising the decomposition of the channel is required, and the omitted channel will be replaced with an extra feature channel to highlight the presence of the vessels across the retina texture.

### Construction of the Feature Map

As discussed through the literature review, the cross-section can be modeled as a Gaussian curve; however, in (10), it was shown that Cauchy PDF (2) could be fitted better than the traditional Gaussian in case, as they are compared in Figure 2.


(2) $$\begin{array}{r} C\left(x\right)=\frac{1}{\pi \gamma \left[1+{\left(\frac{\left(x-x_{0}\right)}{\gamma }\right)}^{2}\right]} \end{array}$$

Where γ and *x* stand for scaling parameter (defining the slope of the curve) and pixel intensity, respectively. By selecting non-zero values for *x*_0_, the curve peak is shifted horizontally. To generate the bank of filters, γ is chosen as 1, the template curves trunk value (*T*) is selected 9, while the length value of each template (*L*) was chosen 8, upon the recommendations for DRIVE dataset in (2).

**Figure 3:**
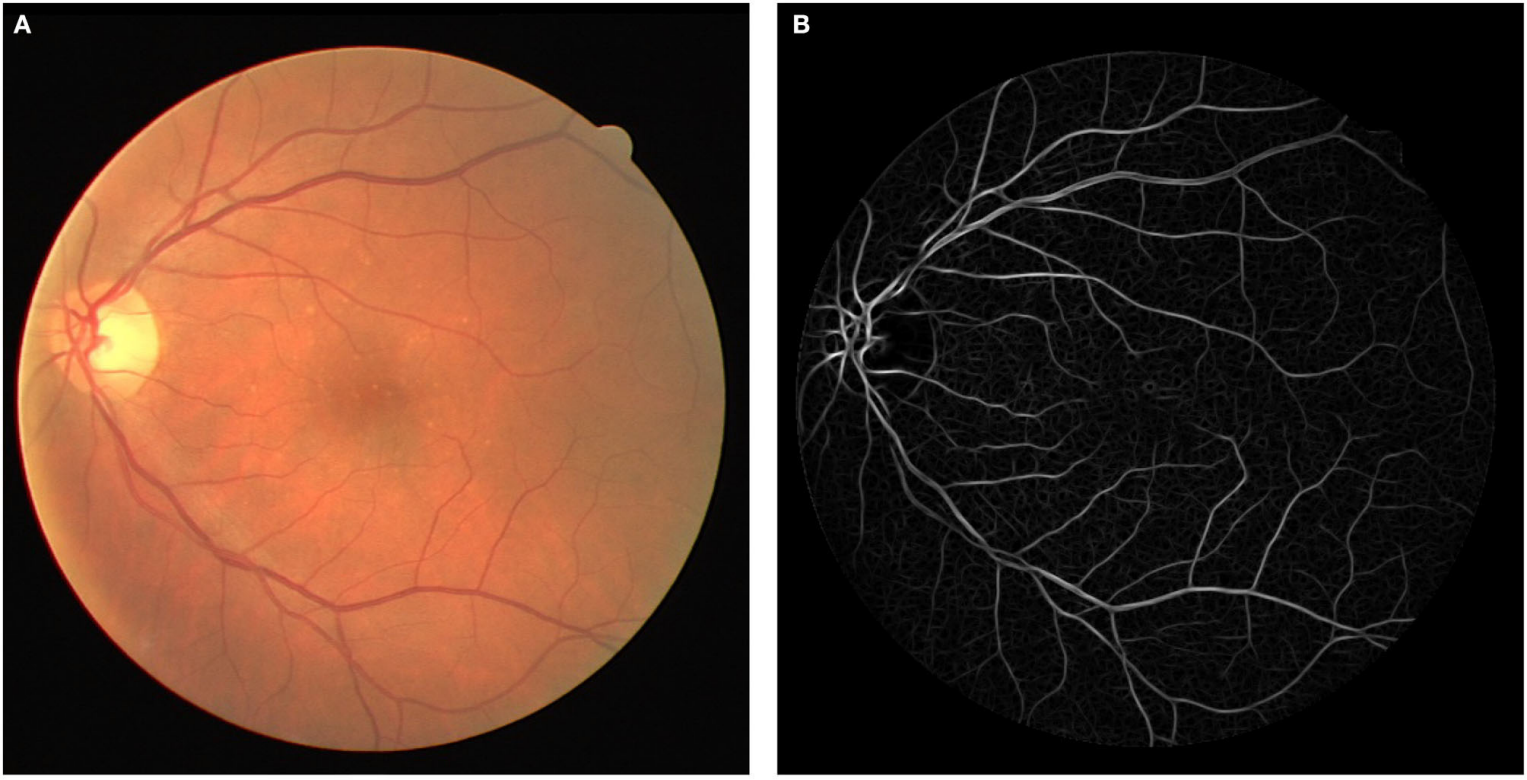
**(A)** Original fundus image and **(B)** Cauchy matched filter response.

The template is then rotated every $\frac{\pi }{12}$ to detect vessels in different orientations. To prepare an image for either training or testing, each pixel is first convolved with the bank of filters, and the highest response is stored. Figure 3 depicts an image with its response after the convolution.

**Figure 4:**
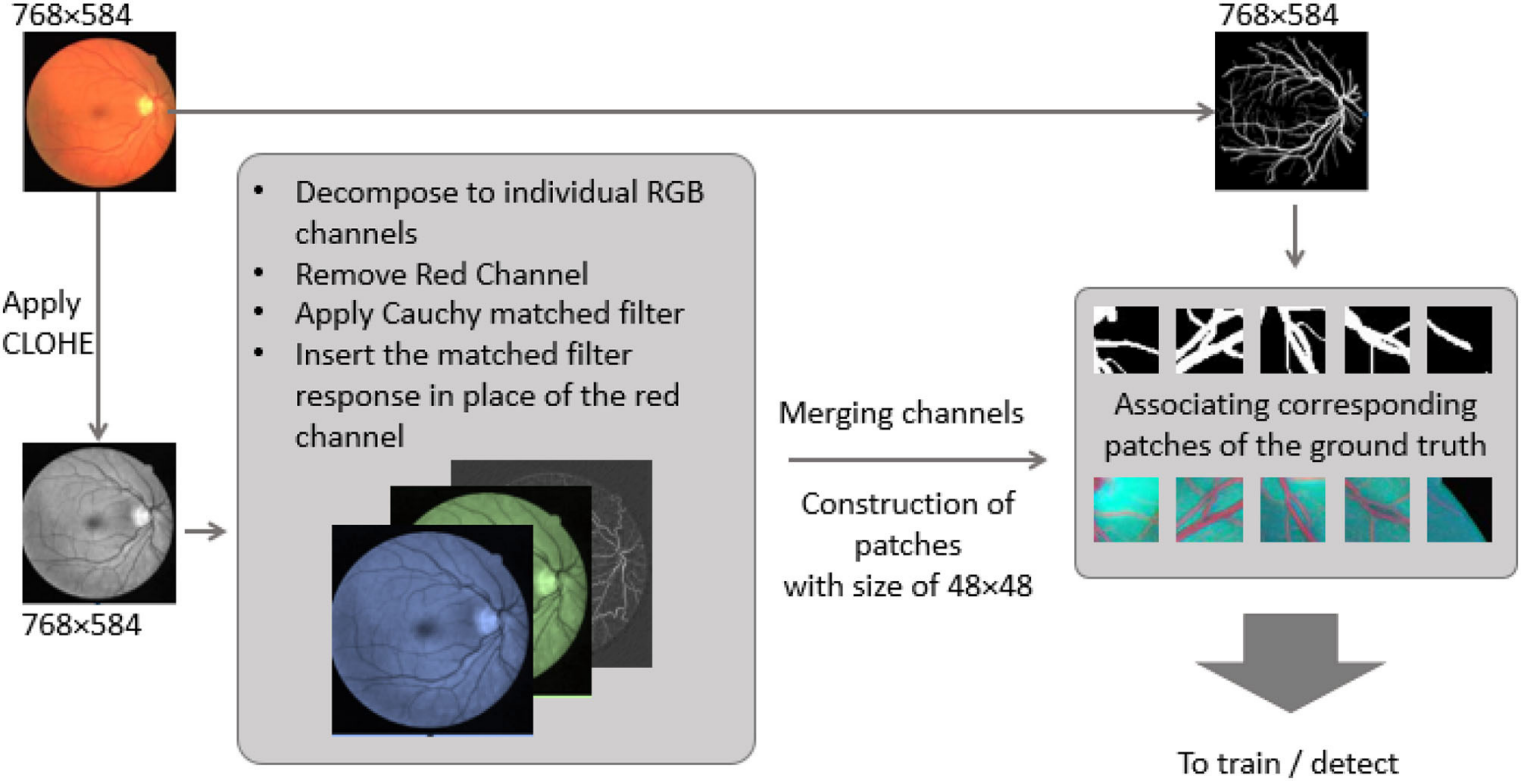
Image preparation pipeline, from original Fundus image to feature map patches.

As the red channel was initially removed from the original image, the matched filter response is inserted in the red channel. To prepare the new feature images (Cauchy-G-B) for creating patches. Since there are few training images (20 for the dataset), a total of 500 overlapped 64 × 64 patches have been generated per training image. Besides, data augmentation has been employed via either flipping patches horizontal and vertically. This operation assured that more than 40k patches are available for the training and validation process. However, for testing purposes, the total ROI of each test image is segmented into 64 × 64 patches with no overlap. It assures that as soon as there is a prediction per each patch, they can be regrouped structurally to form the predicted image entirely. The image preparation pipeline and the patches are presented in Figure 4.

### U-Net

The U-Net is widely employed among the different CNN architectures used for retinal vessel detection (41). U-net has been proven to have a promising output in various segmentation approaches, especially in retinal vessel segmentation (3, 4). Concerning the end-to-end training of pixels, it uses multi-level decomposition to incorporate low-level features and multi-channel filtering. On the one hand, employing U-net enhances training useful low-level features. Yet, on the other hand, through the testing, it discards the trained feature, which results in degradation of learning ability.

**Figure 5:**
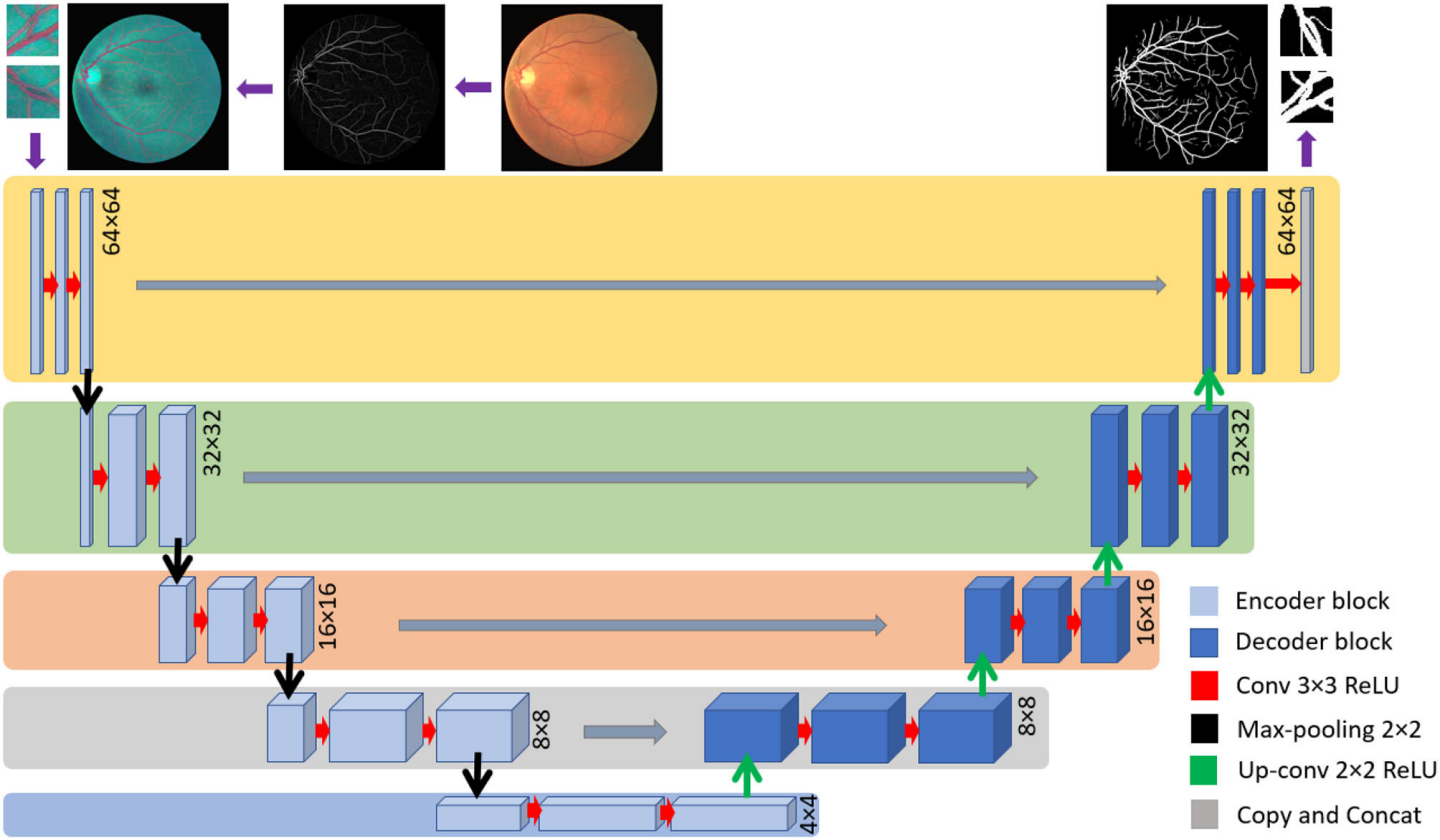
U-net structure implemented in this investigation.

The employed U-net model is based on a fully connected network structure comprising an input layer, a convolutional layer, a pooling layer, and an up-sampling layer followed by the output layer. Bach to the encoding stage includes four down-sampling steps where there are two convolutional layers and one pooling layer in each. The encoding stage consists of four up-sampling steps, while each one consists of two convolutional layers and one up-sampling layer. The input layer is set to accept patches of the size of 64 × 64 × 3, while the output is designed to generate a 64 × 64 × 1 floating point image. The architecture consists of 32 filters with padding for the size equal to the input for the first and second convolutional layers in the internal layers. The Rectified Linear Unit (ReLU) activation function was used in the whole model except the last layer where SoftMax was employed presented in (3).


(3) $$\begin{array}{r} p_{k}\left(x\right)=\frac{exp\left[a_{k}\left(x,y\right)\right]}{\sum_{k^{\prime }=1}^{K}exp\left[a_{k}\left(x,y\right)\right]} \end{array}$$

where *a*_*k*_(*x*) stands for the activation in feature channel *k* at the pixel position of (*x, y*). The number of classes is shown by *K* and *p*_*k*_(*x*) shows the approximated maximum function. The U-net structure is depicted in Figure 5.

## Results and Discussions

### Database

The DRIVE database contains 40 pictures with 768 × 584 pixels captured digitally via a Canon CR5 camera at 45 fields of view (42). Total seven images show some lesions corresponding to degrees of retinopathy. Besides, binary maps containing manual segmentation of vessels are provided, and masks cover retina boundaries. Unfortunately, the images are provided in a lossy compression format (JPEG), which has affected some small details; however, most researchers have used this dataset as a reference for evaluation. The images are split into two groups, training and test, each containing 20 images with three images presenting retinopathy symptoms.

### Implementation Details

We first extracted patches from DRIVE retinal images to train a DL model. Later, we fed them as the input of the designed network and the corresponding label patches obtained from ground truth data. In other words, With the approach used to train a U-net with the end-to-end strategy, the preprocessed patches and their corresponding ground truth extractions were employed to obtain a specific training model for DRIVE database images. Data splitting was carried out by considering 90% of all extracted patches for training and 10% for validation. This strategy has been adopted in several previous studies, including in (42–44), and (33). A total number of 200 epochs were considered for the training process, as well.

#### Training Details

Adam optimizer was employed to speed up the training process and avoid trapping on local minima while training. It included setting β_2_ = 0.9 and β_2_ = 0.999 to handle sparse gradients on noisy (45). While the initial learning rate was set to η = 0.01, and it was set to decrease with the shrinking rate of η_*i*_ = 0.9η_*i*_−1 per each 10 epochs when the value of loss function is saturated.

The system configuration used for the training and testing process includes an Intel Core i7-7300 CPU with 64GB of memory. The code was developed in Python 3.7 using Anaconda and Keras as the DL framework and TensorFlow. The preprocessing, including the preparation of multi-channel feature images, was quite fast per each image (<10 s), but instead, each training epoch required more than 2 h to complete.

### Evaluation Metric

Retinal vessel segmentation is commonly looked at as a binary classification task. The predicted labels for each pixel are compared with the ground truth classes to fit vessel (positive) or retinal tissue (negative) categories. Binary classification requires determining a true or false class (vessel or retinal tissue) class per pixel. Therefore, a predicted pixel can be represented in four groups:

- True Positive (T_P_) are those pixels predicted as vessels and belong to vessels in ground truth images (correct prediction).
- True Negative (T_N_) pixels are predicted as non-vessels and belong to retinal tissue (correct prediction).
- False Positive (F_P_) indicates the predicted pixels as retinal background but instead belong to vessels (incorrect prediction).
- Finally, False Negative (F_N_) are non-vessel pixels, but they have been classified as retinal vessels (incorrect prediction).

For the evaluation purposes, the performance of the trained models is obtained through measuring Sensitivity (Sen), specificity (Spc), accuracy (Acc), and Precision (Prc), which are standard approaches for evaluating binary results represented in (4–7).


(4) $$\begin{array}{r} Acc=\frac{T_{P}+T_{N}}{T_{p}+F_{P}+T_{N}+F_{N}} \end{array}$$

(5) $$\begin{array}{r} Sen=\frac{T_{P}}{T_{p}+F_{N}} \end{array}$$

(6) $$\begin{array}{r} Spc=\frac{T_{N}}{F_{P}+T_{N}} \end{array}$$

(7) $$\begin{array}{r} Prs=\frac{T_{P}}{F_{P}+T_{P}} \end{array}$$

In addition, the area under the Receiver Operating Characteristic curve (AUROC) for comparison with contemporary methods is obtained, as well.

The overall output of the proposed method is obtained via the receiver operating characteristic curve (ROC), which reflects the relationship between true positive and false positive rates concerning different thresholds when the network output is mapped into binary classes. For this purpose, global thresholding method with different threshold values was considered for the threshold-sensitive metrics by calculating sensitivity against the false-positive ratio ($FPR=\;\frac{F_{P}}{F_{N}+T_{P}}$) at various threshold values, and later plotting ROC curve per each FPR and thresholding rate pair. The same strategy is adopted for obtaining the average precision obtained *via* (7) against the threshold at various threshold values.

#### Sensitivity Analysis of Global Threshold

To have robust evaluations for metrics that are sensitive to the employed global thresholding method (Acc, Sen, Spc, and Prc), a range of values has been studied threshold values sampled in τ_*i*_∈{ 0.01, …, 0.99}. The study revealed that the changes in the metrics are slight around τ = 0.5, which shows that this threshold value can be considered to be used for further evaluations.

### Discussion and Comparison With Previous Approaches

Based on the obtained results, it can be discussed that the inclusion of an additional feature channel (the Cauchy matched filter response) improved the detection rate of vessels via enhancing vessel contrast against the background. In contrast, this strategy filtered the noisy red channel. Besides, vessels with various diameters were segmented by having a bank of filters at different scales for the matched filter. Vessels with multiple diameters were segmented, which assisted the DL method to improve the proposed classifier.

**Figure 6:**
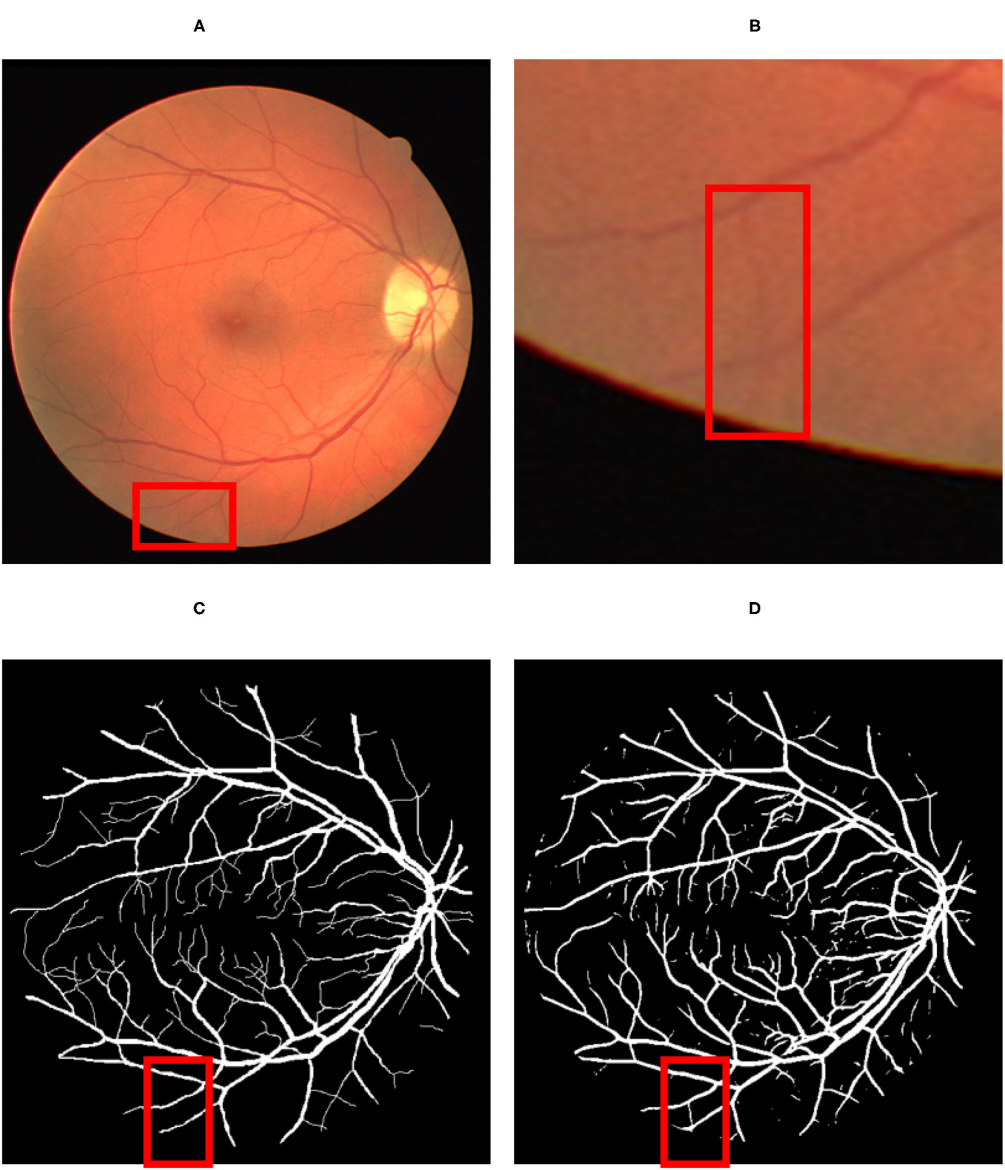
The result of the detection: **(A)** Original fundus image; **(B)** a thin vessel; **(C)** the corresponding ground truth map which does not contain the vessel; **(D)** the thin vessel being detected by the proposed model. The red color square box part in **(A)** has been reflected by respective identified image in **(C)** by proposed map. Similarly the red square part reflected in **(B)** has been identified by red square box part in image **(D)**.

Concerning the subjective characteristic of manual labeling of the ground-truth data, a detailed visual comparison between the predicted vessel map (Figure 6D) and the ground-truth (Figure 6C) reveals that the proposed method can extract more vessels that had not been labeled before in ground truth images (Figures 6A,B).

**Figure 7:**
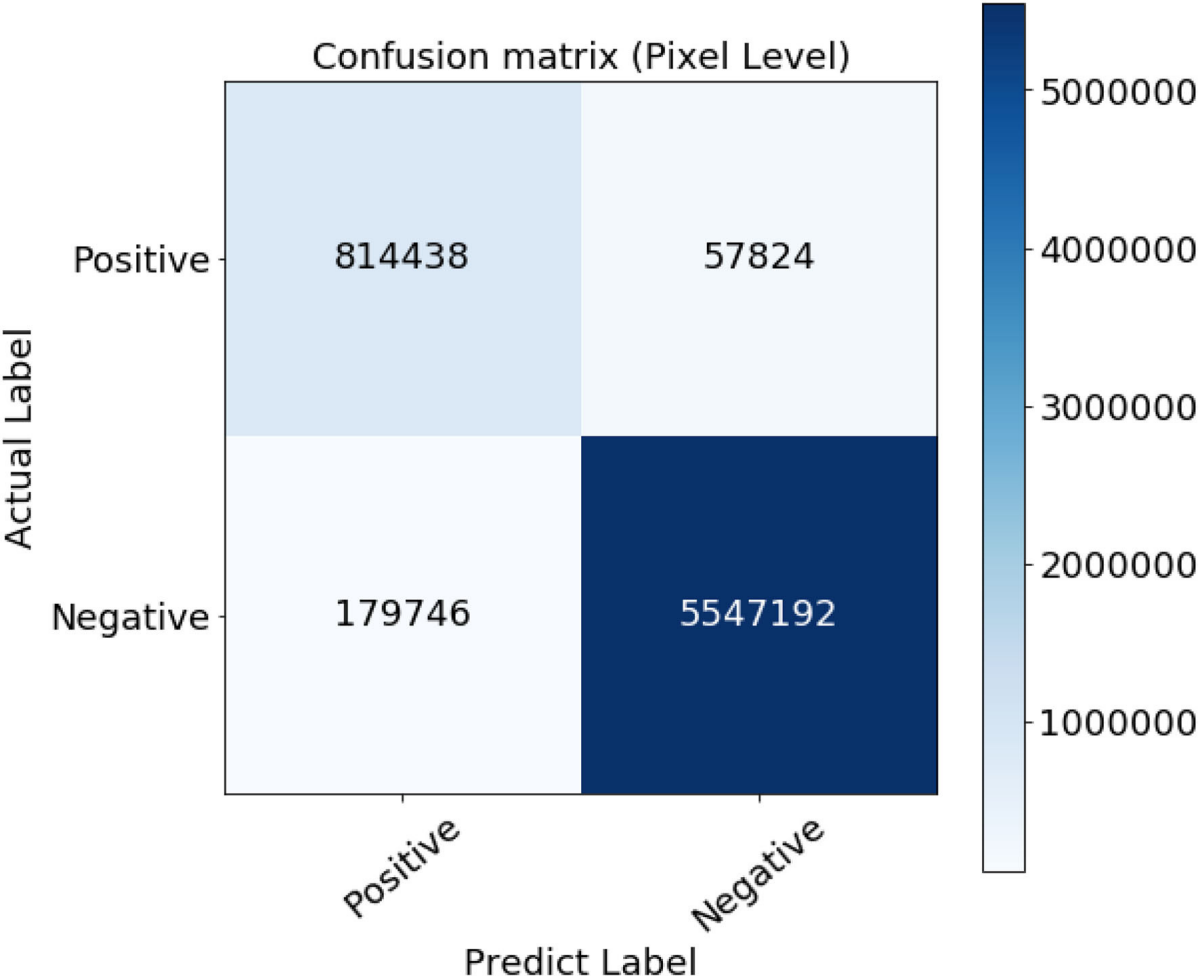
Confusion matrix of the evaluation results.

However, since those new extracted vessels are not included in the subjective extracted ground truth images, total *T*_*P*_ and *T*_*N*_ pixels decreased while *F*_*P*_ and *F*_*N*_ increased, respectively. Such imbalance labeling has impressed the metrics to approach slightly toward a perfect detection. Hopefully, since the ground truth data is common for all the compared methodologies, they can be evaluated simultaneously. The confusion matrix presented in Figure 7 certifies the details of the evaluation.

Additional visual comparison asserts that the proposed methods have detected thinner vessels in the central regions on the retina, where vessels are distributed in low density. It is due to the vessels' shape to fit better on the employed Cauchy curve.

**Figure 8:**
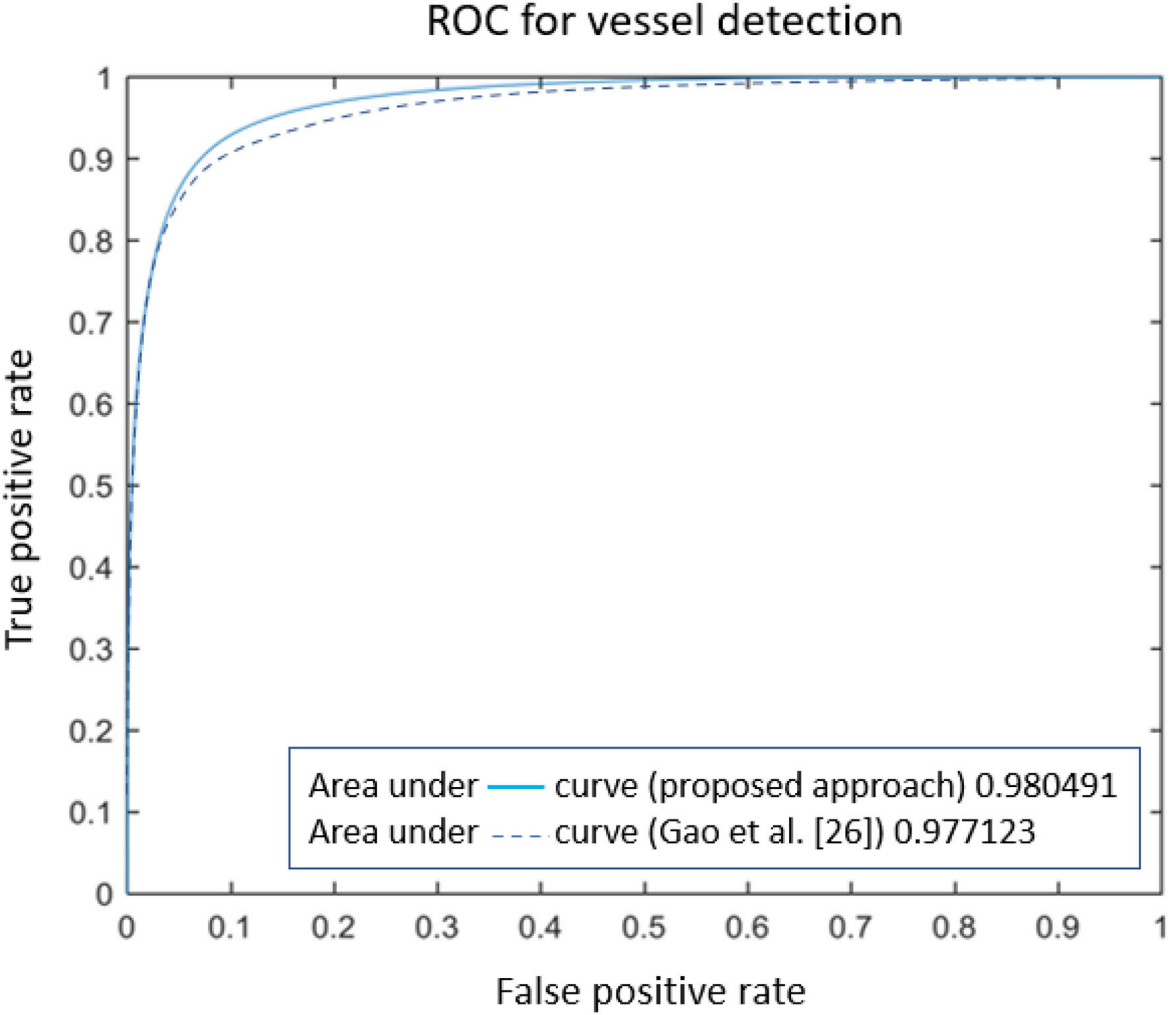
The comparison of ROC curves for retinal vessel detection methods.

Numerical comparison with other approaches (reported in Table 2) certifies the proposed model is ranked among most state-of-the-art methods, based on Sensitivity, Specificity, accuracy, and area under the ROC. However, some methods are ranked higher [such as the one presented in (44)], as they employed octave convolutions to extract multiple-spatial-frequency features aiming to capture retinal vasculatures with different thicknesses. This technique allowed them to improve the U-net structure and, at the same time, take benefit from features to enhance the detection rate significantly. Figure 8 illustrates the AUROC of the proposed method, which shows a bigger area under the curve (0.9805) compared to the baseline model (0.9771) presented by Gao et al. (33).

**Table 2:** Assessment of the obtained results with contemporary approaches.

**Method**	**Acc**	**Sen**	**Spc**	**Prc**	**AUROC**
Fraz et al. (40)	0.9422	0.7302	0.9742	0.9600	0.9502
Nicola et al. (48)	0.9467	0.7731	0.9724	NA	0.9588
Lahiri et al. (24)	0.9480	0.7500	0.9800	NA	0.9500
Melinscak et al. (27)	0.9466	0.7276	0.9785	NA	0.9749
Fu et al. (47)	0.9470	0.7294	NA	NA	0.9523
Gao et al. (33)	0.9636	0.7802	0.7802	0.8948	0.9771
Mou et al. (43)	0.9607	0.8132	0.9783	NA	0.9796
Fan et al. (44)	0.9664	0.8374	0.9790	NA	0.9835
Wang et al. (39)	0.9511	0.7986	0.9736	NA	0.9740
Saroj et al. (46)	0.9509	0.7278	0.9724	NA	0.8501
Proposed method	**0.9640**	**0.8192**	**0.9896**	**0.9337**	**0.9805**

Wang et al. Proposed a model that provides an accuracy rate of 0.9511 (39), while the model proposed by Saroj et al. Shows an accuracy rate of 0.9509 (46). Therefore, it can be concluded that the proposed model's accuracy in this paper is superior to existing models.

## Conclusion and Future Work

Targeting retinal vessel detection, this paper offers a novel approach that pools the response of a traditional matched filter with a fully connected U-net network. The uniqueness of the suggested method is to replace the noisy red channel of fundus images with a channel that can provide more vessel details. It is intended since the red blood vessel are barely visible distributed over the red retina tissue. The new pseudo-color image, called a multi-channel feature map, is processed by generating several patches for training via the U-net. The corresponding labels for the training patches are also provided using the ground truth data. This technique accelerates the training process and provides enough data to train the DL model. Evaluation of the results shows that vessel detection can be accomplished with an accuracy of 96.40% and sensitivity around 82%. This difference is seen since thin vessels have been discarded during the subjective annotation of ground truth images. The blue channel of retinal images can be replaced with a Gabor-filter response in future work. The bank of the used filters can be equipped with vessels presented in a variety of widths. Additional channels are also intended to be investigated to assist the DL model in associating the pixel values to the vessel or retinal tissue classes. Last but not least, and through comparison of this work to the other state-of-the-art methodologies, the proposed method shows a promising result highlighting the importance of combining feature maps to improve the detection of retinal vessels via U-net assisted DL approaches.

Gathering large quantities of fundus images is one of the most perplexing in creating strong deep-learning systems. Designing algorithms that can work from restricted data is one solution. Apart from limited images available in public datasets, there is still an opportunity for testing blood vessel segmentation approaches on noisy real-life pathological retinal pictures. The offered model has been tested only on the DRIVE dataset. Still, it can also be tested on the other available datasets to evaluate its accuracy and compare with other models. Also, the proposed model needs to be tested to real-time pathological retinal images.

## Data Availability Statement

The original contributions presented in the study are included in the article/supplementary material, further inquiries can be directed to the corresponding author.

## Author Contributions

SA and MS: conceptualization, writing—original draft, and preparation. SA, MH, and SB: methodology. SB: validation. MH and FJ: resources. SB, MH, and RA: writing—review and editing and funding acquisition. MS, FJ, and SA: supervision. SA, RA, and SB: project administration. All authors have read and agreed to the published version of the manuscript.

## Funding

This work was supported by the Deanship of Scientific Research, King Faisal University, Saudi Arabia (grant number NA000256).

## Conflict of Interest

The authors declare that the research was conducted in the absence of any commercial or financial relationships that could be construed as a potential conflict of interest.

## Publisher's Note

All claims expressed in this article are solely those of the authors and do not necessarily represent those of their affiliated organizations, or those of the publisher, the editors and the reviewers. Any product that may be evaluated in this article, or claim that may be made by its manufacturer, is not guaranteed or endorsed by the publisher.
